# Treatment of chickens with fluralaner induced mortality in pyrethroid-resistant *Triatoma infestans* (Hemiptera, Triatominae)

**DOI:** 10.1186/s13071-025-07009-1

**Published:** 2025-09-24

**Authors:** Mateo Rocha-Bazán, Alejandra Alvedro, Camila Vázquez-Cañás, Santiago Piñero, Delfina Trezza-Neumayer, Claudia Viviana Vassena, Gustavo Fabián Enriquez, María Sol Gaspe, Marta Victoria Cardinal

**Affiliations:** 1 Facultad de Ciencias Exactas y Naturales Laboratorio de Eco-Epidemiología Intendente Güiraldes 2160, Ciudad Universitaria, Universidad de Buenos Aires, C1428EHA Buenos Aires, Argentina; 2 CONICET-Universidad de Buenos Aires. Instituto de Ecología, Genética y Evolución de Buenos Aires (IEGEBA), Ciudad Universitaria, C1428EHA Buenos Aires, Argentina; 3 Facultad de Ingeniería y Ciencias Exactas, Departamento Agronomía y Ambiente, Universidad Argentina de La Empresa (UADE), Lima 717, Buenos Aires, Argentina; 4 Centro de Investigaciones de Plagas E Insecticidas (CIPEIN)-UNIDEF-CITEDEF-CONICET, San Juan Bautista de La Salle 4397 (CP. 1603), Villa Martelli, Buenos Aires, Argentina

**Keywords:** Xenointoxication, Pyrethroid resistance, Chagas disease, Endectocide

## Abstract

**Background:**

Residual spraying with pyrethroid insecticides is still the main strategy used to prevent vector-borne transmission of *Trypanosoma cruzi*, the etiological agent of Chagas disease. The emergence of resistance to these insecticides in triatomine populations associated with vector control failure highlights the need to evaluate alternative tools, such as xenointoxication. Chickens serve as important blood meal sources and are positively associated with triatomine abundance. Therefore, several endectocides have been tested in chickens, with fluralaner exhibiting the best results. However, the effect of treating chickens with fluralaner has not been evaluated in pyrethroid-resistant triatomines. Here, we aimed to assess the efficacy and duration of the lethal effect of fluralaner on pyrethroid-resistant and susceptible *Triatoma infestans* using chickens as treated hosts under semi-experimental conditions with a treated–control design.

**Methods:**

Three chickens received two oral doses of Bravecto^®^ (fluralaner, MSD Animal Health) at 0.5 mg/kg, whereas three other chickens were assigned to the control group, which received only semolina. Third- to fifth-instar nymphs, both susceptible and resistant to pyrethroid insecticides, were exposed to the chickens at five specific times: 0 (pre-treatment), 3, 7, 14, and 28 days post-treatment (DPT). We recorded the degree of triatomine engorgement and assessed feeding success and survival after each exposure. The data were analyzed via logistic regressions and Kaplan‒Meier curves.

**Results:**

Feeding success rates were high, ranging from 95.5% to 100% throughout the trial, and were not affected by treatment or exposure time. The greatest lethal effects of fluralaner on triatomines exposed to treated chickens were observed up to 14 DPT, with cumulative mortality ranging from 76.9% to 87.0%. At 28 DPT, triatomine mortality decreased significantly to 12.8%, similar to the control group means (< 17.9%) and pre-treatment levels (6.8%). No difference in the lethality of fluralaner was detected between susceptible and pyrethroid-resistant triatomines via logistic regression analysis.

**Conclusions:**

On the basis of these and previous results, chickens are eligible for a field study that addresses the efficacy of simultaneous xenointoxication of various hosts using fluralaner. This approach provides a promising alternative for addressing the challenge of resistance to pyrethroid insecticides in triatomines.

**Graphical Abstract:**

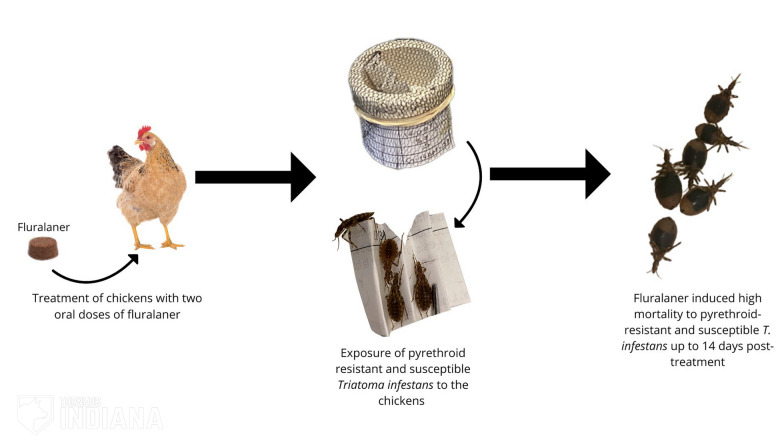

**Supplementary Information:**

The online version contains supplementary material available at 10.1186/s13071-025-07009-1.

## Background

Chagas disease is one of the neglected tropical diseases listed to be controlled as part of the World Health Organization’s 2030 Agenda for achieving the Sustainable Development Goals [1]. A key goal is to interrupt vector-borne transmission in endemic areas, which relies heavily on insecticide spraying, mainly pyrethroids. However, this goal has been hindered by the emergence of pyrethroid resistance in different triatomine species that serve as vectors for *Trypanosoma cruzi* (Kinetoplastida, Trypanosomatidae). For *Triatoma infestans* (Hemiptera, Triatominae), the main domestic vector in the Gran Chaco region, several hotspots of pyrethroid resistance have been described and have caused control failures [2].

An alternative strategy is xenointoxication, the delivery of specific endo- and ectoparasiticides to the vector through their blood meals. Xenointoxication in several host species has been implemented in attempts to control triatomines (reviewed in Tian et al. [3]). Fluralaner showed the most promising results. Xenointoxication with fluralaner using dogs as treated hosts achieved 99–100% mortality of pyrethroid-susceptible and pyrethroid-resistant *T. infestans* in experimental assays for 2–4 months [4, 5]. This strategy reduced the abundance and infestation prevalence of pyrethroid-resistant *T. infestans* in a small field study [6]. Decreases in *T. cruzi* infection prevalence in triatomines as well as in human–vector contact were also observed in that study [7]. Among the few triatomines collected alive in domestic habitats post-treatment, most had fed on cats or chickens, not dogs, whereas in peridomestic ecotopes, chickens were one of the main blood meal sources [7]. Fluralaner-treated chickens achieved mortality rates of 90–100% up to 7 days post-treatment (DPT) in *Triatoma gerstaeckeri*, *T. infestans*, *Triatoma brasiliensis*, *Triatoma pseudomaculata*, and* Rhodnius prolixus* that fed on them [8, 9]. However, whether fluralaner-treated chickens can kill pyrethroid-resistant *T. infestans* has not been addressed. The aim of this study was to assess the efficacy and duration of the lethality of fluralaner on pyrethroid-resistant and pyrethroid-susceptible *T. infestans*, using chickens as treated hosts under semi-experimental conditions with a treatment–control design. We hypothesized that no difference in the lethal effects of fluralaner would be observed between pyrethroid-susceptible and pyrethroid-resistant *T. infestans*.

## Methods

We employed a total of 408 third- to fifth-instar nymphs of *T. infestans*, both susceptible (*n* = 276) and resistant (*n* = 132) to pyrethroids. Colonies of susceptible triatomines were obtained from Santiago del Estero and Chaco provinces in Argentina, whereas those of resistant triatomines were from Mataral, Bolivia [10]. Insects were provided by (i) the Unidad Operativa de Vectores y Ambiente-Centro Nacional de Diagnóstico e Investigación en Endemoepidemias (UnOVE-CeNDIE) ANLIS Malbrán-Ministerio de Salud de la Nación in Punilla, Córdoba, Argentina; (ii) reared in the insectary of the Centro de Investigaciones de Plagas e Insecticidas (CIPEIN); or (iii) obtained from our insectary at the Facultad de Ciencias Exactas y Naturales, Universidad de Buenos Aires (FCEN-UBA). Triatomines were kept in a controlled environment chamber at 26 ± 1 °C, 70% relative humidity, and a 12:12-h light‒dark cycle.

Six chickens [*Gallus gallus domesticus* (Galliformes, Phasianidae)], in a healthy state as evidenced by a general condition score of C2 determined by a veterinarian [11], were housed at the Experimental Field of the FCEN-UBA. Chickens were randomly allocated to the control (*n* = 3) or treatment group (*n* = 3), and each group was maintained in separate enclosures to prevent any potential exposure of untreated chickens to fluralaner residues. In each enclosure, chickens were given food and water ad libitum. Fluralaner was obtained from its commercial formulation Bravecto^®^ (chewable tablet for dogs) and was administered as described previously [8]. Each chicken was weighed with a scale (Pesola^®^), and those in the treated group received two oral doses of 0.5 mg/kg of fluralaner mixed with wheat semolina administered at a 7-day interval [8, 12]. Chickens in the control group were fed semolina only. Triatomines were exposed to each chicken for 20 min, with an additional 10 min if unfed triatomines remained, as described elsewhere [4]. The same six chickens were employed during the trial. The six chickens were exposed at five specific time points: 0 days (before treatment administration) and 3, 7, 14, and 28 DPT. Pyrethroid-susceptible (S) and pyrethroid-resistant (R) triatomines were exposed simultaneously at a 2:1 (S:R) ratio to the same chicken. Each group of triatomines was placed in a separate wooden box covered with tulle mesh, which was held against the ventral surface of the chicken, allowing the insects to feed. After exposure, each group of triatomines was kept in its respective box for the entire observation period. A total of 68 triatomines were used for each chicken, as follows: pre-treatment (*n* = 15), 3 DPT (*n* = 15), 7 DPT (*n* = 12), 14 DPT (*n* = 13), and 28 DPT (*n* = 13). Limited triatomine availability prevented us from using the same number of exposed triatomines and the same distribution of nymph stages in each specific moment. Before exposure to chickens, triatomines fasted for 2–3 weeks. After each exposure, we assessed the feeding success of each triatomine (i.e., whether they had fed or not) and qualitatively classified the engorgement level as unfed, little-fed, medium-fed or fully fed, by transparency, as in Catalá de Montenegro [13]. Triatomine mortality was monitored daily for the first 10 days following each exposure and weekly thereafter, up to 31 days post-exposure.

We defined three different outcomes in triatomines exposed to treated chickens: unaffected (probably unfed insects), dead or moribund, or intoxicated. Dead triatomines consistently displayed a fully extended proboscis, which was not observed in those still showing signs of life. A triatomine was considered moribund when lying on its back with its abdomen and legs facing upward and displaying minimal mobility. Moribund triatomines were pooled with dead triatomines for mortality analysis, as described previously [8, 14]. Intoxicated triatomines showed sublethal effects on locomotion with uncoordinated movements and/or walking difficulties and were pooled with unaffected triatomines in mortality analysis.

### Data analysis

Statistical analyses were conducted using R software (version 4.3.1; R Foundation for Statistical Computing, Vienna, Austria). A logistic regression model was used to evaluate the effects of fluralaner treatment, exposure time, the interaction between fluralaner treatment and exposure time, and pyrethroid resistance status on feeding success using the *lme4* package [15]. The interaction term was not significant and was dropped from the final model. A second logistic regression model was used to evaluate the effects of pyrethroid resistance status, fluralaner treatment, exposure time, and the interaction between these last two variables on survival, with each chicken included as a random effect. Given that the interaction term in this regression was significant, we obtained the estimated marginal means and derived the odds ratios (ORs) and corresponding 95% confidence intervals (CIs) via the *emmeans* package [16]. We set the “resistant” category as the reference level because pyrethroid-resistant *T. infestans* may exhibit increased feeding success [17]. The global fit of the logistic regression models to the data was analyzed via the Hosmer–Lemeshow test implemented within the package *ResourceSelection* [18]. Triatomine survival throughout the trial was analyzed via Kaplan–Meier curves implemented with the *survival* [19] and *survminer* [20] packages and compared with the log-rank test via the *survival* package. We considered the cumulative mortality over the 31 days post-exposure. A significance threshold of *P* < 0.05 was used for all statistical analyses.

## Results

Chickens treated with fluralaner exhibited no adverse effects, and all chickens maintained the general condition score C2 throughout the experimental period as assessed by a trained veterinarian.

Triatomine feeding success rates ranged from 95.5% to 100% throughout the trial, with a mean of 96.9% ± 0.02% (mean ± standard deviation). Qualitative assessment revealed that the most frequent levels of engorgement achieved were “medium-fed” and “fully fed”. The percentage of medium-fed triatomines ranged from 43.6% to 50.6%, whereas the percentage of fully fed triatomines ranged from 23.6% to 41.0% at 0–14 DPT. At 28 DPT, all of the exposed triatomines had successfully fed, and 46.2% of triatomines had fed to repletion (Additional File 1, Fig. S1). Logistic regression analysis indicated that feeding success was not associated with treatment or exposure time (Table 1, Additional File 1, Fig. S1). Pyrethroid resistance seemed to modify feeding success, and the OR of a susceptible triatomine having a successful feeding was 0.15 (95% CI 0.01–0.78), although this result was only marginally significant (*P* = 0.07; Table 1, Additional File 1, Fig. S1). The model adequately fit the data (Hosmer–Lemeshow test, *χ*^2^ = 4.57, *df* = 8, *P* = 0.80).

**Table 1:** Logistic regression analysis of potential factors affecting feeding success: fluralaner treatment, exposure time (in days), and pyrethroid resistance

Explanatory variables	Levels in model	Odds ratio	95% CI	*P*-value
Group	Control	Reference		
	Treatment	1.18	0.38–3.76	0.77
Exposure time	Pre-treatment	Reference		
	3 DPT	1.03	0.23–4.53	0.96
	7 DPT	1.66	0.31–12.33	0.56
	14 DPT	1.11	0.23 – 5.83	0.89
	28 DPT^a^	1.90 × 10^11^	0.00–inf	0.99
Resistance status	Resistant	Reference		
	Susceptible	0.15	0.01–0.78	0.07

*DPT* days post-treatment

^a^All triatomines fed at this exposure time

**Fig. 1:**
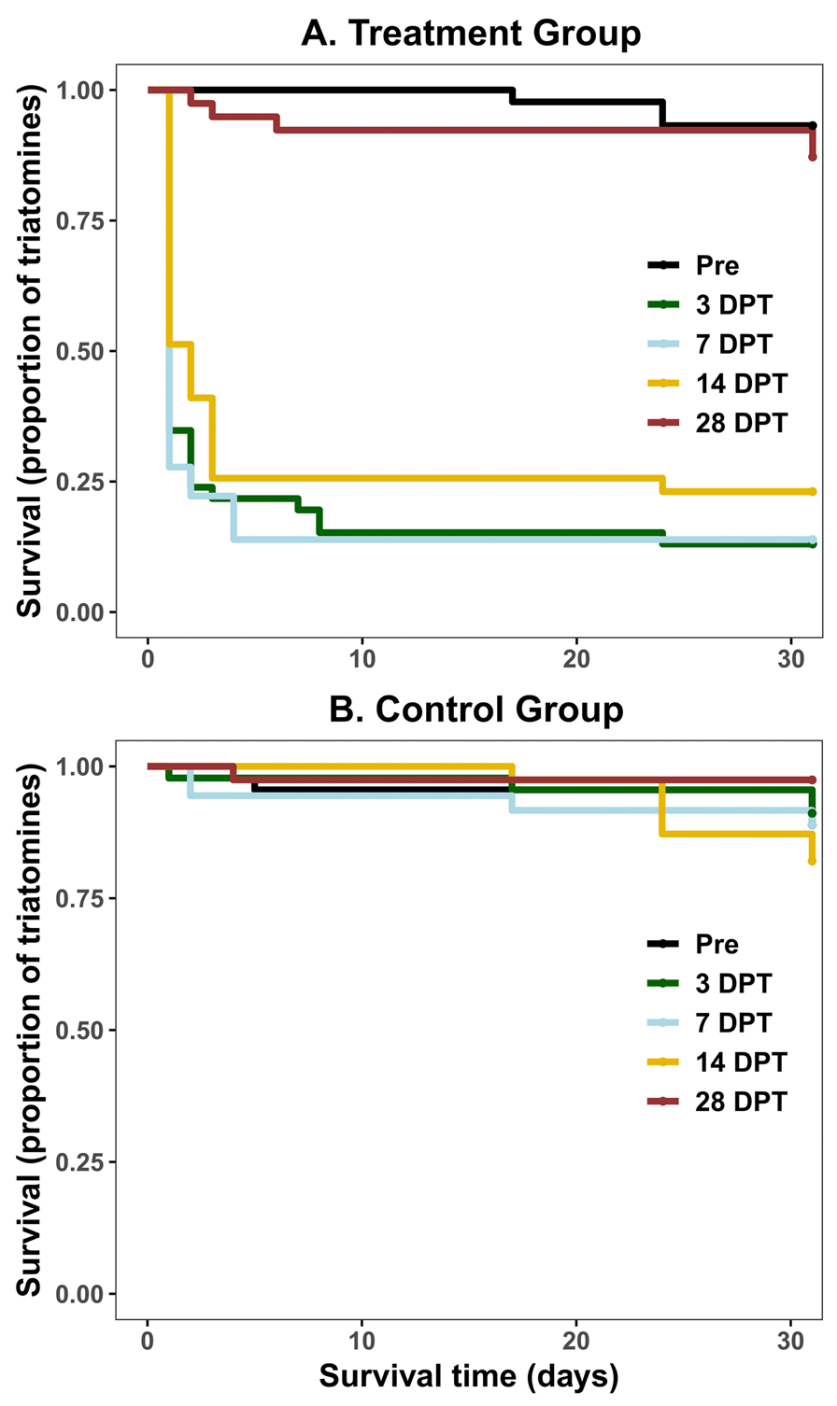
Kaplan‒Meier survival curves for triatomines exposed to fluralaner-treated chickens (**A**) and control chickens (**B**)

Fluralaner exhibited a significant lethal effect up to 14 DPT and sublethal effects on locomotion up to 28 DPT. Some of the initially intoxicated triatomines were able to recover (Additional File 2, Fig. S2), particularly insects exposed at 28 DPT (Additional File 2, Fig. S2D). The cumulative mortality rate reached 86.1% and 87.0% in triatomines that fed on treated chickens at 3 and 7 DPT, respectively, but decreased to 76.9% at 14 DPT (Fig. 1A). Triatomine mortality decreased significantly at 28 DPT to 12.8%, with mortality rates similar to pre-treatment levels (6.8%). No significant difference was detected between the survival curves for the treated and control groups at 0 and 28 DPT (log-rank test, pre-treatment: *χ*^2^ = 0.5, *df* = 1, *P* = 0.5; 28 DPT: *χ*^*2*^ = 2.8, *df* = 1, *P* = 0.09) (Fig. 1A). The cumulative mortality of triatomines exposed to the control group ranged from 2.6% to 17.9% across the five exposure times (Fig. 1B). By means of logistic regression with random effects for chickens, we revealed a significant interaction effect between fluralaner treatment and exposure time at 3, 7, and 14 DPT on triatomine mortality. No association of pyrethroid resistance status with mortality was observed (Table 2). The model adequately fit the data (Hosmer–Lemeshow test, *χ*^2^ = 10.38, *df* = 8, *P* = 0.24).

**Table 2:** Logistic regression analysis of potential factors affecting triatomine mortality: fluralaner treatment, exposure time (in days), and their interaction, and pyrethroid resistance status

Explanatory variables	Levels in model	Odds ratio	95% CI	*P*-value
Group	Control	Reference		
	Treatment	0.81	0.18–3.66	0.78
Exposure time	Pre-treatment	Reference		
	3 DPT	0.52	0.14–1.93	0.33
	7 DPT	0.67	0.18–2.52	0.55
	14 DPT	1.22	0.38–3.91	0.74
	28 DPT	0.13	0.02–1.12	0.06
Resistance status	Resistant	Reference		
	Susceptible	1.53	0.80–2.91	0.20
Group × Exposure time	Control × Pre-treatment	Reference		
	Treatment × 3 DPT	71.56	14.63–349.92	< 0.01
	Treatment × 7 DPT	49.85	9.71–255.96	< 0.01
	Treatment × 14 DPT	15.71	3.83–64.42	< 0.01
	Treatment × 28 DPT	5.57	0.52–58.86	0.13

*DPT* Days post-treatment

## Discussion

Our study demonstrated a lethal effect of fluralaner administered to chickens on pyrethroid-resistant and pyrethroid-susceptible *T. infestans,* with cumulative mortality ranging from 76.9% to 87.0% up to 14 DPT. These results support the use of fluralaner in a multi-host systemic insecticide treatment strategy (i.e., xenointoxication) to cope with pyrethroid resistance in triatomine vectors in a pilot field study. Xenointoxication takes advantage of the usual hosts acting as blood meal sources for triatomines (and other blood-feeding arthropods) to maximize the control of disease vectors while simultaneously minimizing effects on non-target, beneficial insects that would have been impacted by the residual spraying of insecticides [21].

Chickens and domestic dogs are the main non-human blood meal sources of (peri)domestic *T. infestans* [7, 22, 23]. The presence of these animals inside domiciles affects human–vector contact and, therefore, *T. cruzi* transmission risks [23, 24]. Xenointoxication by treating dogs with fluralaner not only reduced *T. infestans* abundance and infestation but also decreased *T. cruzi* infection in the vector and the proportion of human-fed insects [7] in a rural area with triatomine populations exhibiting high levels of pyrethroid resistance [25]. In our current study, fluralaner induced the highest mortality (86–87%) in susceptible and pyrethroid-resistant *T. infestans* exposed to treated chickens between 3 and 7 DPT. Interestingly, the observed mortality at 14 DPT (i.e., 77%) when *T. infestans* and Bravecto^®^ as the source of fluralaner were employed was intermediate between that of *T. gerstaeckeri* (50%) at the same time point [8] and the mortality observed (> 91%) in *R. prolixus*, *T. infestans*, *T. pseudomaculata*, and *T. brasiliensis* when Exzolt^®^ was used as the source of fluralaner [9]. Whether the different sources of fluralaner, methodological issues, or vector species can explain these mortality differences remains to be established. A much longer lethal effect was observed when dogs were treated with fluralaner compared to chickens [4, 5, 13, 26]; however, the recommended dose of fluralaner per kilogram may explain this difference.

Between 3 and 14 DPT, most (74.4–86.1%) of the insects exposed to treated chickens died within the first 120 h, as was also reported when dogs were treated with fluralaner [5, 13]. This is a relevant feature when treating mammalian hosts considering that the time needed for metacyclogenesis after an infective blood meal is estimated to be 5 days in *T. infestans* [27]. The occurrence of sublethal effects and recovery of intoxicated triatomines observed at 28 DPT was surprising. Whether these intoxicated insects may survive in real-life contexts where free-roaming poultry and other birds may prey on them is unknown. However, this observed recovery of intoxicated insects may indicate the time frame for re-treatment with fluralaner to avoid the potential survival of exposed insects, which favors eventual tolerance/resistance mechanisms. Further research is needed to elucidate the best timing (between 14 and 28 DPT) for re-treatment. The withdrawal times for eggs and meat in fluralaner-treated chickens are 0 and 14 days, respectively, as reported by the manufacturer. Fourteen days is coincident with the reported duration of fluralaner detection in chicken plasma by liquid chromatography–tandem mass spectrometry [8]. Treating and re-treating chickens in real-life scenarios would require safety measures to prevent the consumption of treated animals before their withdrawal time.

The occurrence of anti-saliva antibodies to *T. infestans* (and other triatomines) in chickens exposed to repeated triatomine feeding events [28] was postulated as a possible cause of reduced or altered blood intake [8]. However, in our study, feeding success was not associated with exposure time, although repeated feedings were performed on the same chicken. Feeding success and engorgement levels were not associated with the administration of fluralaner, as reported previously when treating dogs [4, 5, 26] and chickens [8, 9], a desirable feature for the use of fluralaner in a xenointoxication strategy.

Our study has two main limitations. First, a limited sample size precluded a more precise estimation of the predictor effects or comparisons among insect developmental stages. Second, maintaining both fed and unfed triatomines together after each exposure limited our capacity to disentangle fluralaner effects from starvation.

## Conclusions

Our results support the use of fluralaner for xenointoxication to cope with pyrethroid-resistant *T. infestans*. Multi-host treatment with fluralaner including chickens seems to be a promising vector control tool that could interrupt vector-borne *T. cruzi* transmission in these scenarios. New studies are needed to address the scalability and cost-effectiveness of this integrated strategy.

## Supplementary Information

Additional file 1. Fig. S1. Engorgement level of triatomines at the five exposure times classified by pyrethroid resistance status, S: susceptible, R: resistant, DPT: days post-treatment.

Additional file 2. Fig. S2. Proportion of dead, intoxicated and live triatomines after exposure to fluralaner-treated chickens (A, 3 DPT, days post-treatment; B, 7 DPT; C, 14 DPT; D, 28 DPT). The dotted red line in each graph represents the proportion of unfed triatomines corresponding to each specific time, and therefore, the minimum proportion of triatomines expected to survive.

Additional file 3. Table S1. Database

## Data Availability

Data are provided within the manuscript or supplementary information files.

## Abbreviations

CI: Confidence interval
DPT: Days post-treatment
OR: Odds ratio
