# Supplementary figures and images for: Treatment of chickens with fluralaner induced mortality in pyrethroid-resistant Triatoma infestans (Hemiptera, Triatominae)
Source: Parasit Vectors. 2025 Sep 24;18:384. doi: 10.1186/s13071-025-07009-1 (PMC12462107; doi:10.1186/s13071-025-07009-1)

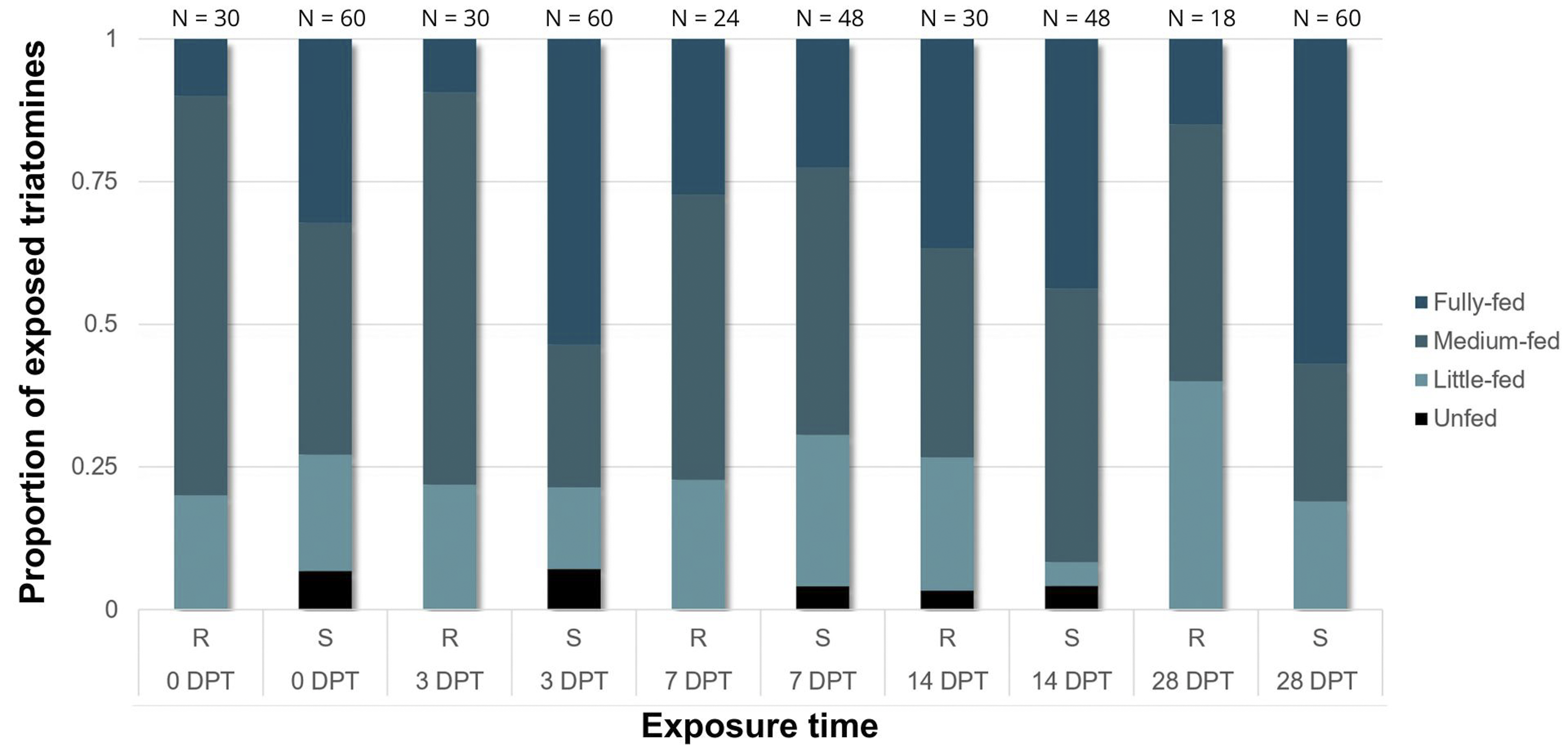

Supplement: Supplementary file 1 — Additional file 1. Fig. S1. Engorgement level of triatomines at the five exposure times classified by pyrethroid resistance status, S: susceptible, R: resistant, DPT: days post-treatment. [file 13071_2025_7009_MOESM1_ESM.tif]

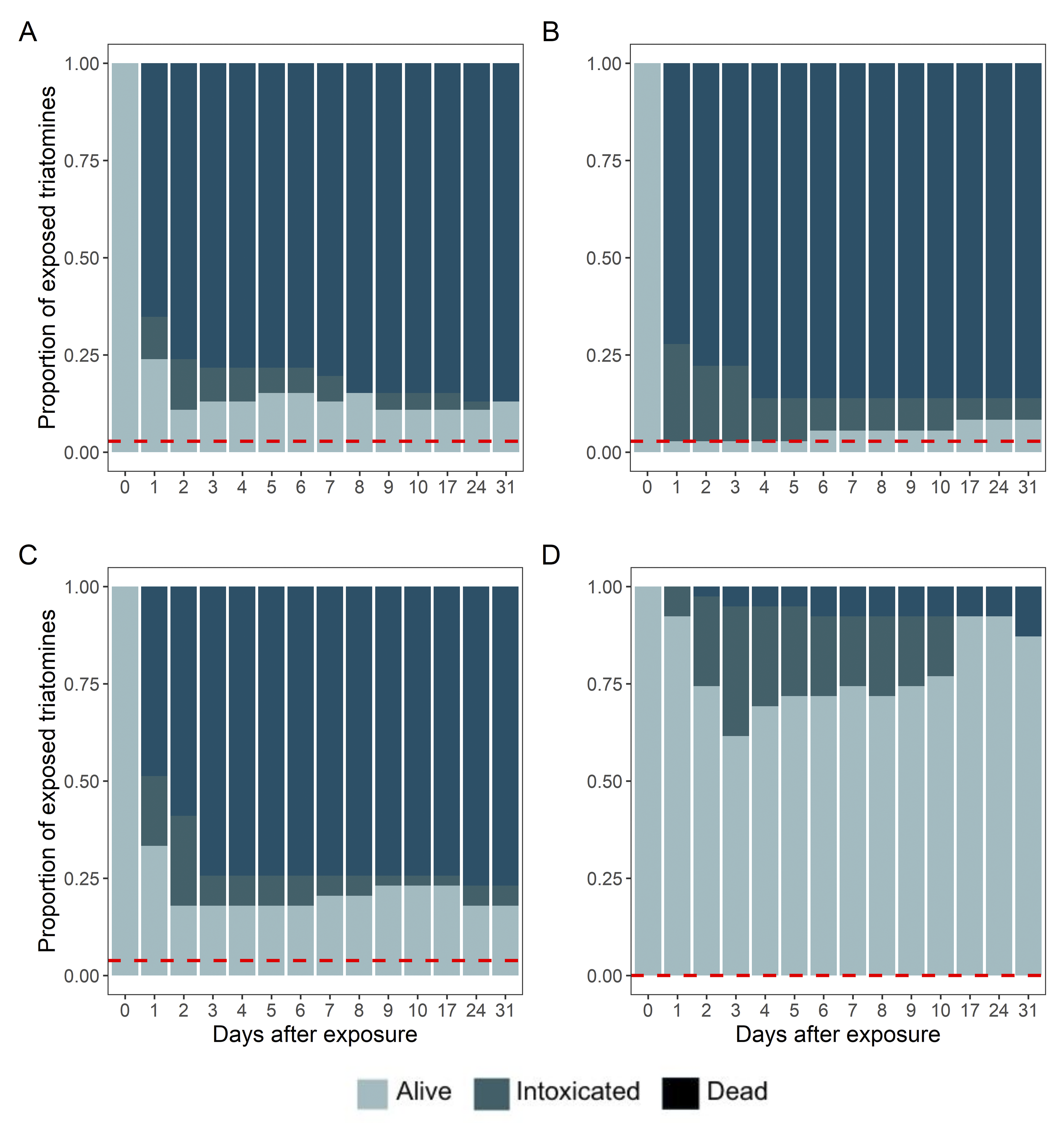

Supplement: Supplementary file 2 — Additional file 2. Fig. S2. Proportion of dead, intoxicated and live triatomines after exposure to fluralaner-treated chickens (A, 3 DPT, days post-treatment; B, 7 DPT; C, 14 DPT; D, 28 DPT). The dotted red line in each graph represents the proportion of unfed triatomines corresponding to each specific time, and therefore, the minimum proportion of triatomines expected to survive. [file 13071_2025_7009_MOESM2_ESM.tif]
